# Effectiveness of collaborative treatment using Korean and Western medicine for mild cognitive impairment or dementia: A protocol for a prospective observational exploratory study

**DOI:** 10.1097/MD.0000000000012098

**Published:** 2018-08-21

**Authors:** Hye-Yoon Lee, Hyung-Won Kang, NamKwen Kim, Eun-Hye Hyun, Joo-Hee Seo, Yeoung-Su Lyu, In Chul Jung, Geun-Woo Kim, Bora Park, Sung-Youl Choi, Hye-Won Kim, Hyun-Min Kim

**Affiliations:** a Pusan National University Korean Medicine Hospital, Yangsan-si, Gyeongsangnam-do; b Wonkwang University Sanbon Hospital, Gunpo-si, Gyeonggi-do; c School of Korean medicine, Pusan National University, Yangsan-si, Gyeongsangnam-do; d National Medical Center, Jung-gu, Seoul; e Department of Korean Neuropsychiatry Medicine, College of Korean Medicine, Wonkwang University, Iksan-si, Jeollabuk-do; f Department of Oriental Neuropsychiatry, College of Korean Medicine, Daejeon University, Daejeon; g Department of Neuropsychiatry, Dongguk University Bundang Oriental Hospital, Seongnamsi, Gyeonggi-do; h Gwangju Oriental Hospital of Wonkwang University, Nam-gu, Gwangju; i Department of Neuropsychiatry, College of Korean Medicine, Gachon University, Seongnam-si, Gyeonggi-do, Republic of Korea.

**Keywords:** collaborative treatment, dementia, mild cognitive impairment, pilot project of Korean medicine-Western medicine collaborative treatments, traditional Korean medicine

## Abstract

**Background:**

South Korea has a dual medical system comprising conventional Western medicine (WM) and traditional Korean medicine (KM), which has yielded both positive results (increased opportunity to choose medical care) and negative results (increased medical costs). Thus, the Ministry of Health and Welfare has been performing a pilot project to evaluate this collaborative system in the real clinical situation. As treatment of dementia requires a social approach, the Korean government aims to strengthen the role of the national health care system to reduce the burden of dementia. The aim of this study was to evaluate the clinical - and cost-effectiveness of collaborative KM and WM treatment in patients with dementia or mild cognitive impairment (MCI) in Korea.

**Method/design:**

In total, 180 patients with dementia or MCI will be recruited and will undergo monthly check-up for 12 weeks. Information regarding demographic characteristics, baseline disease-related data, and outcomes related to cognitive function and quality of life will be obtained. For data analysis, the patients will be classified into 2 groups using a comparative observational study design: the sole treatment group, which will receive either WM or KM alone, and the collaborative treatment group, which will receive both WM and KM.

**Discussion:**

The treatment of dementia/MCI in South Korea will be studied in the real world during the pilot project. There will be no limitations on the type of treatment or the specific treatment method. Examining the clinical- and cost- effectiveness of the different methods will supply information for building an optimal medical system for the treatment of dementia/MCI.

**Trial registration:**

The protocol for this study has been registered at the clinical research information service (CRIS: KCT0002868).

## 1 Introduction

The Republic of Korea has a dualized medical system, established in 1951, which consists of Western medicine (WM) and Korean medicine (KM). The dualized system increases the opportunity for each patient to choose the medical care that he or she hopes to receive and helps to preserve traditional knowledge. However, it has some negative aspects, such as conflicts between practitioners of the 2 systems, and duplicated medical use.^[1]^ Academic studies and institutional discussions have been carried out to address these issues, and the necessity of collaborative treatment wherein WM and KM exist in an independent system and are used cooperatively has been suggested.^[2]^

Factors hindering collaborative treatment include lack of experience and knowledge of practitioners of one system regarding the other system^[3–5]^ and the limitations of the regulatory and legal systems, including the National Health Insurance System (NHIS).^[3,6–8]^ The NHIS of Korea currently covers only the cost of previous care if patients receive both WM and KM for the same disease in the same day. As this practice was recognized as an obstacle to collaborative treatment, the Korean government conducted a “first-stage pilot project of KM-WM collaborative treatments,” which also covered the cost of subsequent treatment by the NHIS.^[9]^ This pilot project yielded positive results, such as increased doctor and patient satisfaction and a decrease in the length of treatment.^[10]^ This initial pilot project was followed by “the second-stage pilot project of KM-WM collaborative treatments,” which is ongoing since November 2017. The second-stage pilot project emphasizes cooperation and consultation between medical doctors and Korean medical doctors (KMDs) according to an optimized protocol for each disease. It also allows for additional costs called “WM-KM consultation fees” for diseases that were frequently observed in the first-stage pilot project or considered important diseases in Korea based on prevalence and mortality.

Prevention, early detection, active treatment, and long-term management including social support are important in the treatment of dementia,^[11]^ which is one of the “4 major diseases” in South Korea.^[12]^ The Korean government is aiming to strengthen the national approach to dementia using programs such as the “national responsibility for dementia care.”^[13,14]^ Mild cognitive impairment (MCI) is an intermediate condition between normal aging and the initial state of dementia. In this condition, the degree of deterioration of cognitive function is mild and there are no deficits in the ability to perform activities of daily living (ADL). MCI is also recognized as a predictor of future progression to dementia.^[15,16]^ Previous studies have reported the effectiveness of KM treatment for cognitive dysfunction,^[17–19]^ and KM treatments are widely used in South Korea for treatment of cognitive impairment.^[20]^

We would like to determine whether collaborative treatment of dementia/MCI using both WM and KM is more effective and cost-effective than treatment using either WM or KM. We have designed a prospective, multicenter, observational study investigating cognitive function, quality of life (QoL), and medical use parameters such as cost and duration.

## 2 Methods and design

### 2.1 Study design and setting

**Figure 1:**
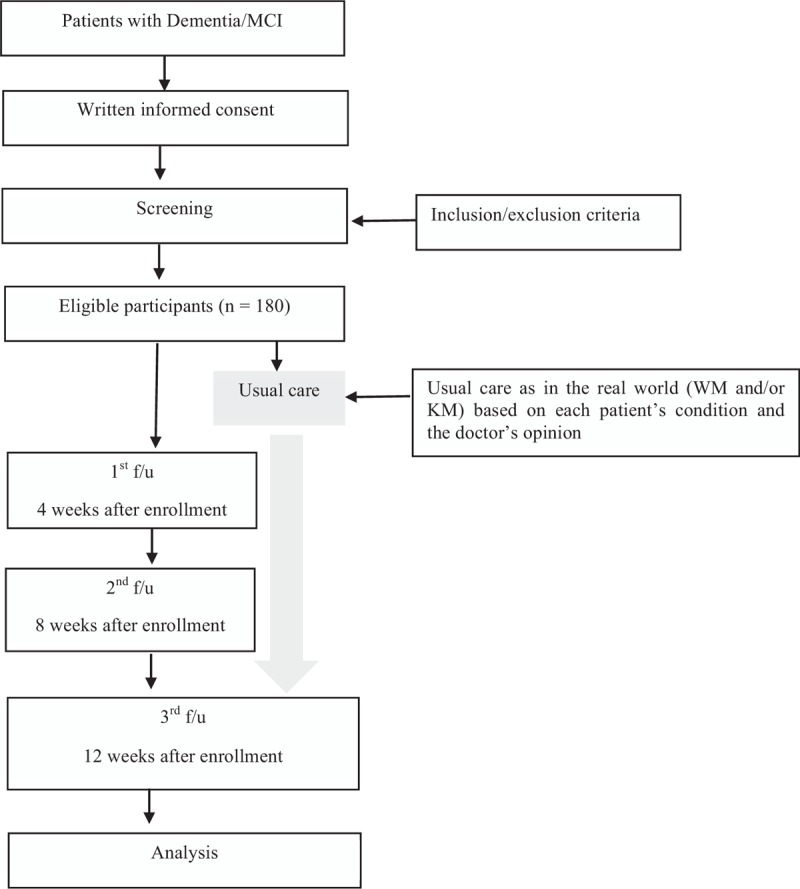
Flow diagram. f/u = followup, KM = Korean medicine, MCI = mild cognitive impairment, WM = Western medicine.

A multicenter, outpatient department (OPD)-based, prospective observational study of patients diagnosed with MCI or dementia is being conducted from April 2018 to December 2018, as a part of a Registry for Korean Medicine and Western Medicine Collaborative Treatment (REKOMENT study). Participants are recruited from 6 university-affiliated hospitals and 1 national medical center, which are participating in the “second-stage pilot project of KM-WM collaborative treatments.” The participants receive treatments from practitioners of KM and/or WM as they would in the real world, and are asked to undergo additional assessments. We will analyze the type of treatment (collaborative or sole treatment), changes in assessment scores, and costs for treatment of MCI/dementia. A comparative observational study design has been adopted to compare the participants in a collaborative treatment (CT) group and those in a sole treatment (ST) group. The study flowchart can be found in the flow diagram (Fig. 1).

### 2.2 Participants

#### 2.2.1 Inclusion criteria

1. Adult patients from institutions participating in the “second-stage pilot project of KM-WM collaborative treatment” whose main diagnosis is MCI or dementia (10th revision of the International Statistical Classification of Diseases and Related Health Problems F00, F01, F02, F03, F067, G30, or G31) aged 75 years or younger.
2. Patients whose global Clinical Dementia Rating (CDR) score is between 0.5 and 2.0 (0.5 ≤ CDR ≤ 2.0).
3. Patients who decide to participate in the study by their own voluntary will (or that of a legal representative when the patient has limited ability to agree, but can still express willingness to participate) and submit written consent.

#### 2.2.2 Exclusion criteria

1. Patients who are currently participating in another clinical trial
2. Patients who have difficulties in participating in the study, as judged by the researchers, such as
  - Those who are expected to have difficulty in complying with the study schedule.
  - Those who are expected to have difficulty understanding and responding to the study questionnaires, such as those who are illiterate or those with serious health conditions (e.g., unstable vital signs).

### 2.3 Consent and registration

The subjects are recruited from the OPDs of 7 of the hospitals participating in the pilot project. Independent researchers who are not in charge of the treatment of the subjects inquire regarding the willingness of the subjects to participate in this observational study. The researchers provide the potential subjects with detailed written information regarding the study and provide them with the opportunity to ask questions regarding the study. The researchers emphasize that the patient's participation in this study will not have any effect on his or her treatment. When a subject finally decides to participate in this observational study, he or she will be asked to submit written informed consent. For patients with a limited ability to consent, an explanation of the study will be provided to both the patient and the patient's legal representative and written consent will be obtained from both individuals. In this case, the study will proceed to the next step only when the patient certainly expresses his/her willingness to participate.

After providing written consent, the subjects will be screened for the eligibility criteria. When a subject is judged suitable for participation in the study, he or she will be registered for the study and assigned a registration number.

### 2.4 Data sources, measurements, and management

Data regarding effectiveness will be collected using participant surveys and clinical evaluations. Data regarding costs will be obtained using participant surveys, administrative data from each institution, and Health Insurance Review and Assessment (HIRA) data.

The participant survey includes information regarding demographic characteristics, medical history, use of long-term care services, and costs for the MCI/dementia treatments. The demographic information obtained includes date of birth, sex, level of education, and occupation (current or past). The medical history information collected includes treatment history, other diseases that the patient has, history of drinking and smoking, and family history.

The Korean Medical Pattern Identification for Dementia (KPD) score will be used to assess pattern identification in each patient.^[21]^ Pattern identification is also known as syndrome diagnosis, and is a major method used to classify symptoms. It is broadly used in traditional Chinese medicine and KM. Information regarding long-term care services will also be obtained, including the score of long-term care insurance service and the contents of long-term care service currently in use. Direct medical costs and direct nonmedical costs of the MCI/dementia treatments will be assessed using surveys of patients or their guardians based on the “limited societal perspective.”^[22]^

The clinical evaluation includes: assessment of the severity of MCI/dementia using measures such as the CDR,^[16]^ Global Deterioration Scale (GDS),^[23]^ Mini-Mental State Exam (MMSE),^[24]^ Korean Montreal Cognitive Assessment (MoCA-K),^[25]^ and Short-Form Geriatric Depression Scale (S-GDpS);^[26]^ and 2) assessment of QoL using measures such as the Seoul Instrumental Activities of Daily Living (S-IADL),^[27]^ Neuropsychiatric Inventory-Questionnaire (NPI-Q),^[28]^ Barthel Activities of Daily Living (Barthel-ADL),^[29]^ EuroQoL-5 Dimension (EQ-5D),^[30]^ and EuroQol-visual analog scale (EQ-VAS).^[31]^

Appropriate standardization procedures have been implemented to minimize differences between evaluators in the different institutions as much as possible. KM or WM physicians with >1 year of clinical experience are in charge of the clinical evaluations. The QoL assessment is based on responses of patients or their guardians to a questionnaire. The patients and/or guardians are allowed to ask the researcher questions if there are difficulties in understanding the meaning of a question. However, they are required to respond to the questions independently without intervention from the researchers.

Administrative data from each institution will be used to determine the costs of MCI/dementia treatments, and the type and frequency of collaborative treatment. Data obtained from the HIRA include medical expenses of the patients, the duration of medical use, and the type of medical services used by the patients.

The time schedule for data collection is presented in Table 1. All collected data will be recorded using iCReaT, which is a type of electronic case report form supplied by the Korea National Institute of Health. The study procedures including the data collection and management will be monitored by the Monitoring Center for Korean Medicine and Western Medicine Collaboration, which is governed by the Ministry of Health and Welfare.

**Table 1:**
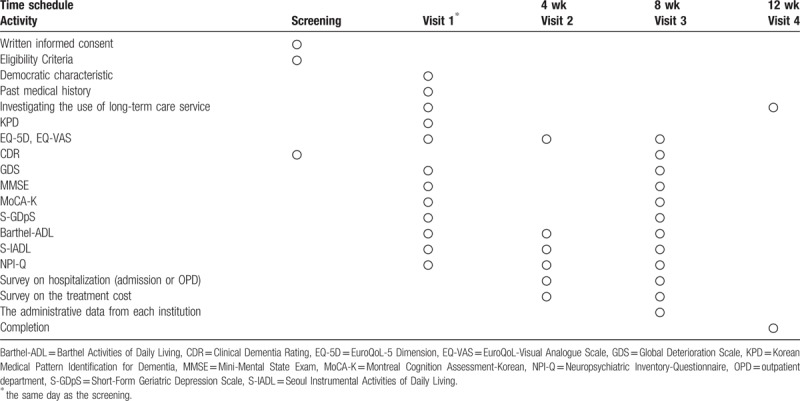
Data collection schedule.

### 2.5 Outcome measures

#### 2.5.1 Primary outcomes

##### 2.5.1.1 QoL

Changes in S-IADL, Barthel-ADL, NPI-Q, and S-GDpS scores from baseline to 4 and 8 weeks after the baseline will be used to evaluate effectiveness in terms of QoL.

The S-IADL consists of 15 questions scored from 0 to 3, wherein higher scores indicate more disability in ADL. The questionnaire evaluates current and potential performance separately.^[27]^

The Barthel-ADL is widely used to evaluate ADL because it requires a short time and is easy to score.^[32]^ There are various modifications of the test, including one by Collin et al^[33]^, who simplified the scoring system to range from 1 to 20. The Korean version of the Barthel-ADL has been shown to be valid and reliable.^[29]^

The NPI-Q is used to measure abnormal behaviors. It is read and evaluated by caregivers. The questionnaire can be easily used in the medical field in the real world.^[28]^ The official Korean version of the NPI-Q is used in this study. This version is distributed by the Mapi Research Trust (https://eprovide.mapi-trust.org).

The S-GDpS is used to evaluate the symptoms of depressive mood in elderly individuals. It consists of 15 questions. The Korean version of the S-GDpS is used in this study.^[26]^

##### 2.5.1.2 Cognitive function

Changes in the MoCA-K, MMSE, CDR, and GDS scores from baseline to 8 weeks will be used to evaluate effectiveness in terms of improvement in cognitive function.

The MoCA was created to screen for individuals with MCI, who have normal scores on the MMSE. The questionnaire consists of visuospatial/executive ability, naming, memory, attention, language, abstraction, delayed recall, and orientation domains.^[34]^ The MoCA-K, which is a validated Korean version of the test,^[25]^ is used in this study. We have registered the study on *mocatest.org*.

The MMSE is one of the most broadly used screening tests for dementia. It was created by Folstein et al and can be completed in 5 to 15 minutes. This test has a total score of 30. It has the advantage of being useful in assessing changes over time, based on repeated measures during disease progression. This is because the test only has a small learning effect.^[35]^ The reliability and validity of the MMSE have been demonstrated in distinguishing moderate and severe dementia.^[24]^ The official Korean version of the test, which is supplied by the Psychological Assessment Resources, is used here.

The CDR is the most widely used test to evaluate the severity of dementia. It is divided into the following domains: memory, orientation, judgment and problem-solving, community affairs, home and hobbies, and personal care. Each category is scored on a 5-point severity scale (none, questionable, mild, moderate, and severe).^[36]^ The Korean CDR has been shown to be valid.^[16]^ The score is determined by the doctor, who makes his or her assessment based on detailed examinations of the patient and his or her caregiver.

The GDS is used to evaluate overall cognitive function. It is scored on a scale of 1 to 7 (0, no cognitive decline; 2, very mild cognitive decline; 3, mild cognitive decline; 4, moderate cognitive decline; 5, moderately severe cognitive decline; 6, severe cognitive decline; and 7, very severe cognitive decline).^[37–39]^ The validity of the Korean version of the GDS has been demonstrated.^[23]^

##### 2.5.1.3 Others

“Long-term care score” and “use of long-term care service” will be investigated at baseline and 12 weeks after the baseline. The “use of long-term care service” includes home-visit care, home-visit bathing, home-visit nursing, day and night care, short-term care, and use of geriatric care facilities.

#### 2.5.2 Secondary outcomes

The secondary outcomes in our study comprise cost-effectiveness analysis measurements. Cost components such as direct medical costs, and direct nonmedical costs will be measured using case report forms and hospital administrative data. The preference-based health utility measure instrument of the EQ-5D-5L will be calculated as quality-adjusted life years using the area under the curve method.^[40]^ The incremental cost-effectiveness ratio (ICER),^[41]^ the confidence interval of the ICER, and the cost-effectiveness acceptability curve (CEAC)^[42]^ will also be analyzed to evaluate the significance of the cost-effective outcomes in South Korea. The schedule for data collection can be found in Table 1.

### 2.6 Treatments

The participants receive treatment for MCI/dementia, mostly based on their practitioners’ clinical opinions; thus, there is no fixed treatment for the study. The participants can freely choose any type of WM and/or KM treatment method. When a participant wishes to receive both WM and KM treatment, the practitioner of 1 type of medicine used for the patient's treatment will make a “request for a collaborative treatment” to a practitioner of the other type of medicine. The participants can freely decide the type of treatment in consultation with their practitioners at any time, as with general care. All treatments that the participants receive will be recorded and monitored.

### 2.7 Rationale for sample size

The aim of this study is to collect complete data from 120 patients assuming adequate recruitment during the study period. Estimating a 20% missing data rate, a minimum of 150 patients should be enrolled. Thus, considering the inherent characteristics of a prospective observational study, 180 participants will be recruited to collect sufficient data for statistical analysis and interpretation.

### 2.8 Statistical and analytical plans

Baseline characteristics and disease progression will be compared between the CT and ST groups. All continuous variables will be tested to confirm normality. Student *t* tests or Wilcoxon rank sum tests will then be used to compare the means of the 2 groups. Categorical data will be analyzed using *χ*^2^ or Fisher exact tests. Per-protocol and intention-to-treat (ITT) analyses will be used to assess all effectiveness and cost-effectiveness data. Missing volume, pattern, and mechanisms will be analyzed before the ITT analysis. If the missing mechanism is identified as missing completely at random or missing at random, as defined by Rubin,^[43]^ the multiple imputation method will be used to replicate the missing data. Scores on the S-IADL, Barthel-ADL, S-GDpS, NPI-Q, MoCA-K, MMSE, CDR, GDS, and QoL as measured by the EQ-5D and EQ-VAS, and the use of long-term care service will be compared between the 2 groups for effectiveness analysis. Means and 95% confidence intervals for continuous variables and frequencies and percentages for categorical variables will also be calculated. All statistical analyses will be performed using 2-tailed tests at a significance level of .05. In the economic evaluation, the ICER results will be analyzed deterministically using mean values, and the statistical significance of differences in ICER will be determined using the bootstrapping method. CEAC will also be analyzed and reported along with the meaningful ranges for the national thresholds. Stata MP version 14 (StataCorp LLC, TX) and SAS version 9.4 (SAS Institute Inc., Cary, NC) will be used for all of the aformentioned analyses.

### 2.9 Ethics and dissemination

This study has been approved by the institutional review boards (IRBs) of National Medical Center (H-1803–088–002), Wonkwang University Sanbon Hospital (WMCSB 201802-11), Dongguk University Bundang Oriental Hospital (DUBHIRB2018-0001), Gil Korean Medicine Hospital of Gachon University (18-103), Wonkwang University Jeonju Korean Medicine Hospital (WUJKMH-IRB-2018-0001), Gwangju Oriental Hospital of Wonkwang University (2018/3), and Daejeon University Dunsan Korean Medicine Hospital (DJDSKH-18-BM-05). The protocol for this study has been registered at the clinical research information service (KCT0002868).

A KMD will ask participants who have decided to voluntarily enroll in the study to submit written consent. All data obtained from the participants will be secured. Hard copies will be stored in double-locked locations and soft copies will be saved in memory devices not connected to the internet also stored in double-locked locations. The corresponding author will have access to the final dataset. All participants will be protected and respected according to the Declaration of Helsinki and related laws and regulations. The present findings will be disseminated in a report published in a peer-reviewed journal.

## 3 Discussion

This protocol for a study with a prospective observational design has been developed to study the real-world clinical situation. As such, it does not restrict the type of intervention used and instead allows the use of any type of intervention or combination of interventions.

The present study has some limitations. First, the study is conducted in the OPD setting because the pilot project targets OPD patients alone. As a result, information regarding patients with severe dementia that requires hospitalization will not be obtained, and the data obtained in this study cannot be generalized to all dementia patients. Second, the institutions participating in this study are those that are participating in the government “pilot project.” These institutions are relatively well-equipped for collaborative treatment, and thus do not represent the current status of collaborative treatment in all clinical institutions in Korea. Lastly, the observation period is short (12 weeks) because of the nature of the pilot project. Thus, there may be difficulties with obtaining sufficient data to encompass the natural progression and treatment process. However, we believe that the data related to effectiveness and cost-effectiveness obtained in this study can be used as basic information and reference data for future studies. Furthermore, this study is the first to evaluate the effects of collaborative treatment on dementia/MCI at the national level in Korea, and will help build the ideal medical care system for dementia/MCI in concordance with the movement to strengthen the national responsibility for dementia.

## Author contributions

Lee, Kang, and NK Kim conceived the research question and designed the study. Hyun, HW Kim, and HM Kim planned the specific steps of the study. Seo, Jung, Park, Lyu, and KW Kim contributed to the design of the study and review of the manuscript as clinical experts. Lee drafted the manuscript. NK Kim critically reviewed the overall manuscript and takes full responsibility for the study. All authors have read and approved the final manuscript.

**Conceptualization:** Hye-Yoon Lee, Hyung-Won Kang, NamKwen Kim.

**Methodology:** Hye-Yoon Lee, Hyung-Won Kang, NamKwen Kim, Eun-Hye Hyun, Joo-Hee Seo, Yeoung-Su Lyu, In Chul Jung, Geun-Woo Kim, Bora Park, Sung-Youl Choi, Hye-Won Kim, Hyun-Min Kim.

**Project administration:** Hye-Yoon Lee, Hyung-Won Kang, NamKwen Kim.

**Supervision:** NamKwen Kim.

**Writing – original draft:** Hye-Yoon Lee.

**Writing – review & editing:** NamKwen Kim.

Author name: 0000-0002-9486-1703.
